# Arthroscopic Partial Trapeziectomy and Free Tendon Suspension and Interposition Combined with Internal Brace for Basal Joint Arthritis of Thumb

**DOI:** 10.3390/jcm14124118

**Published:** 2025-06-10

**Authors:** Kuang-Ting Yeh, Jen-Hung Wang, Jochieh Li, Jui-Tien Shih

**Affiliations:** 1 Department of Orthopedics, Hualien Tzu Chi Hospital, Buddhist Tzu Chi Medical Foundation, Hualien 970473, Taiwan; micrograft@tzuchi.com.tw; 2 School of Medicine, Tzu Chi University, Hualien 970374, Taiwan; 3 Institute of Medical Sciences, Tzu Chi University, Hualien 970374, Taiwan; 4 Graduate Institute of Clinical Pharmacy, Tzu Chi University, Hualien 970374, Taiwan; 5 Department of Medical Research, Hualien Tzu Chi Hospital, Buddhist Tzu Chi Medical Foundation, Hualien 970473, Taiwan; paulwang@tzuchi.com.tw; 6 Department of Orthopaedic Surgery, Taoyuan Armed Forces General Hospital, Taoyuan 325208, Taiwan; orthojochieh@aftygh.gov.tw

**Keywords:** carpometacarpal thumb arthritis, arthroscopic partial trapeziectomy, joint arthritis

## Abstract

**Background:** Carpometacarpal thumb arthritis causes pain and functional limitations. **Methods**: This study evaluated the efficacy of arthroscopic partial trapeziectomy with free palmar longus tendon suspension and interpositional arthroplasty, combined with a soft anchor internal brace, for the treatment of thumb basal joint arthritis. Between August 2010 and April 2020, 60 thumbs with symptomatic basal joint arthritis (Eaton stage II–III) were treated using this minimally invasive technique. **Results**: The cohort included 52 female and 8 male patients (mean age, 62.6 ± 4.3 years), who underwent clinical follow-up for 28.7 ± 3.0 months. VAS pain scores decreased from 5.7 ± 0.5 to 1.0 ± 0.7 and 7.1 ± 0.6 to 1.4 ± 0.9 (*p* < 0.001) during rest and activity, respectively. Thumb range of motion increased from 43.3 ± 11.3 to 54.2 ± 9.8 degrees, while pinch strength improved from 47.3 ± 9.5% to 88.8 ± 17.3% of the contralateral side (*p* < 0.001). Patients with Eaton stage II disease demonstrated better outcomes than those with stage III disease. Radiographically, minimal proximal migration of the first metacarpal (mean, 1.2 mm) was observed, with no cases of scaphotrapezial joint arthritis. **Conclusions**: Arthroscopic partial trapeziectomy with tendon suspension/interposition and an internal brace is an effective treatment for Eaton stage II–III basal joint arthritis, offering significant pain relief, functional improvement, and joint stability, while preserving the scaphotrapezial joint.

## 1. Introduction

Carpometacarpal (CMC) thumb arthritis is one of the most common types of upper limb arthritis. Typical conservative treatment for this condition comprises activity modification, medication, injections, and splinting. Currently, the most common surgical treatment used is trapezium excision combined with ligament reconstruction and tendon interposition (LRTI) [1]. While a total trapeziectomy with LRTI has been considered the gold standard for treating advanced basal joint arthritis, partial trapeziectomy offers several potential advantages. First, preserving a portion of the trapezium maintains the osseous foundation of the thumb, potentially providing better stability and preventing excessive proximal migration of the first metacarpal. Second, partial trapeziectomy minimizes the risk of damage to the scaphotrapezial (STT) joint, which can be a source of persistent pain following total trapeziectomy. Third, by preserving the proximal portion of the trapezium, the procedure maintains the integrity of important ligamentous attachments that contribute to overall joint stability. Finally, the arthroscopic approach to partial trapeziectomy allows for a less invasive procedure with potentially faster recovery times and less postoperative pain compared to open total trapeziectomy techniques [2,3,4]. Traditional LRTI typically involves an open surgical approach with complete excision of the trapezium, followed by harvesting a strip of the flexor carpi radialis (FCR) tendon while maintaining its distal attachment. This tendon is then passed through a drill hole in the base of the first metacarpal and secured, with the remaining tendon folded and placed into the void created by the trapezium’s removal. Our arthroscopic technique offers several key improvements: (1) preservation of the proximal trapezium and STT joint, reducing the risk of proximal metacarpal migration and maintaining the thumb’s length; (2) a minimally invasive approach with smaller incisions, potentially reducing postoperative pain and accelerating rehabilitation; (3) addition of an internal brace to enhance stability; and (4) thermal shrinkage of attenuated ligaments to improve joint congruity. These modifications aim to address the limitations of traditional LRTI while maintaining its established benefits. Advances in arthroscopic technology allow the examination and treatment of small joints throughout the body with minimal morbidity. Synovitis, osteophytes, ligamentous tears, and laxity were successfully managed with arthroscopic surgery. Moreover, arthroscopy is reliable for the direct evaluation and treatment of the thumb CMC joints [5].

We hypothesized that in patients with symptomatic basal joint arthritis, arthroscopic resection of the distal one-third of the trapezium combined with thermal shrinkage of the volar ligaments and arthroscopic LRTI, along with soft anchor internal bracing and a temporary K-wire fixation, would enhance suspension stability. We analyzed the clinical results over a minimum follow-up period of 2 years.

## 2. Materials and Methods

The study was conducted in accordance with the Declaration of Helsinki and ap-proved by the Institutional Review Board II of the Tri-Service General Hospital (protocol code: TSGHIRB No.: 2-107-05-154 and date of approval: 17 December 2018). Informed consent was obtained from all subjects involved in the study. From August 2010 to April 2018, 60 consecutive patients (60 thumbs) with symptomatic basal joint arthritis (Eaton stage II–III) underwent arthroscopic partial trapeziectomy with tendon suspension and interposition arthroplasty, combined with internal brace augmentation. All patients had failed conservative treatment for at least 6 months, including rest, thumb bracing, activity modification, nonsteroidal anti-inflammatory medication, and corticosteroid injections (mean: 2 injections, range: 1–6). 

**Figure 1:**
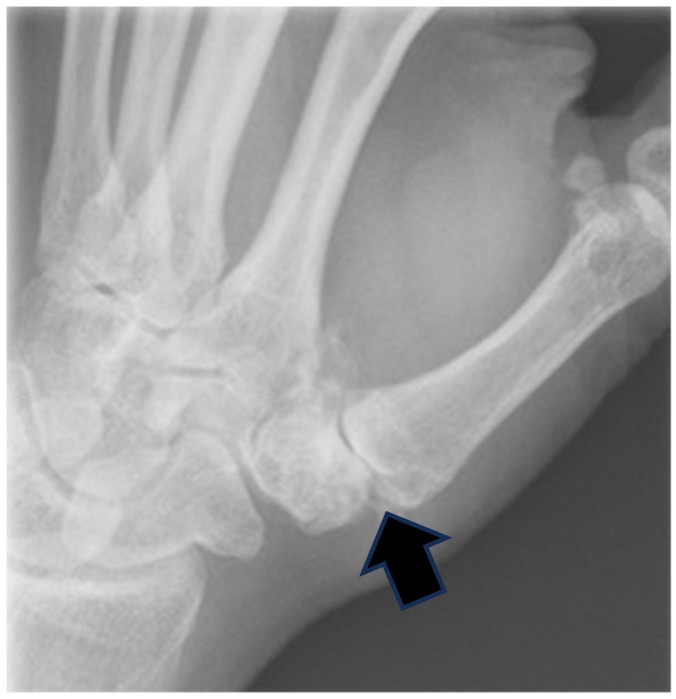
Preoperative radiograph of a 68-year-old female with Eaton stage III basal joint arthritis. The black arrow indicates the collapsed first carpometacarpal joint with osteophyte formation and subchondral sclerosis.

Patients were included in this study if they had radiographic stage II or III basal joint disease according to the Eaton and Glickel classification [6], presented with symptomatic disease despite adequate conservative management, and had a minimum 2-year follow-up available for analysis (Figure 1). The physical examination included assessment of thumb basal joint range of motion, tenderness localization, axial stress grading test, and thumb pinch strength measurement using a Jamar dynamometer. Plain radiographs of the thumbs were obtained from all patients to confirm the diagnosis and staging. Patients were excluded from the study if they had concurrent thumb injuries, other concomitant regional diagnoses that could confound the interpretation of results such as Bennett’s fracture or de Quervain’s tenosynovitis, or incomplete follow-up data. Additionally, patients with previous surgical interventions on the affected thumb or those with systemic inflammatory arthropathies were excluded to ensure a homogeneous study population focused specifically on primary osteoarthritis of the carpometacarpal joint.

### 2.1. Surgical Technique

**Figure 2:**
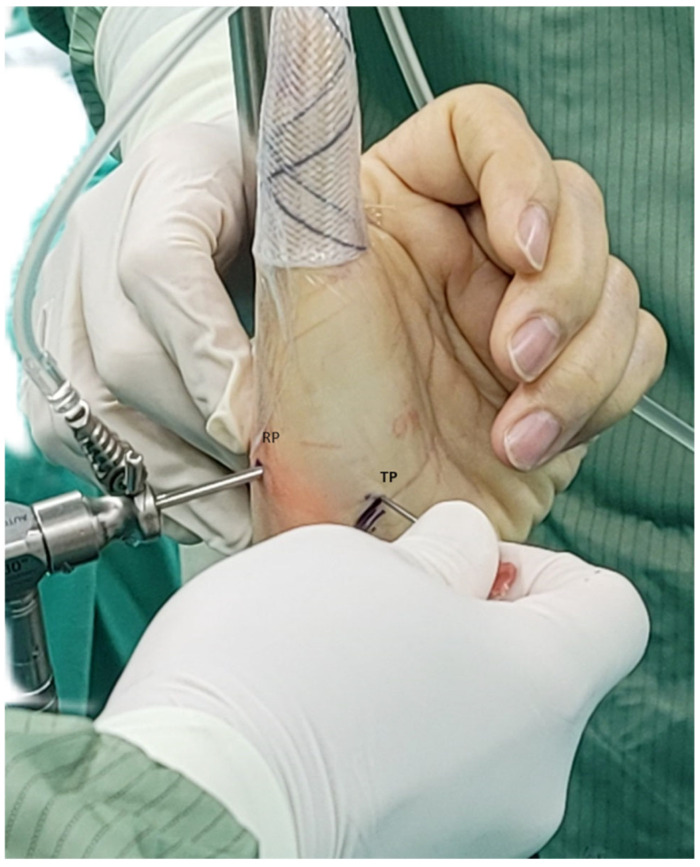
Radial portal (RP) created over the radial border of APL, followed by the creation of the thenar portal (TP) over the FCR tendon region.

All procedures were performed under general anesthesia with tourniquet control. The thumb was placed in a single-finger trap with 5–10 lb of longitudinal traction using a Linvatec traction device. Two arthroscopic portals were established following the technique described by Berger [5]: (1). Radial portal (RP): Located between the abductor pollicis longus (APL) and extensor pollicis brevis (EPB) tendons for visualization of joint cartilage and ligamentous structures; (2) Thenar portal (TP): Positioned over the flexor carpi radialis (FCR) tendon region in the thenar muscle area for assessment of dorsal ligaments (Figure 2). Joint distension was achieved through traction and injection of 2–3 mL normal saline to facilitate insertion of a 1.9-mm, 30° short-barreled arthroscope.

**Figure 3:**
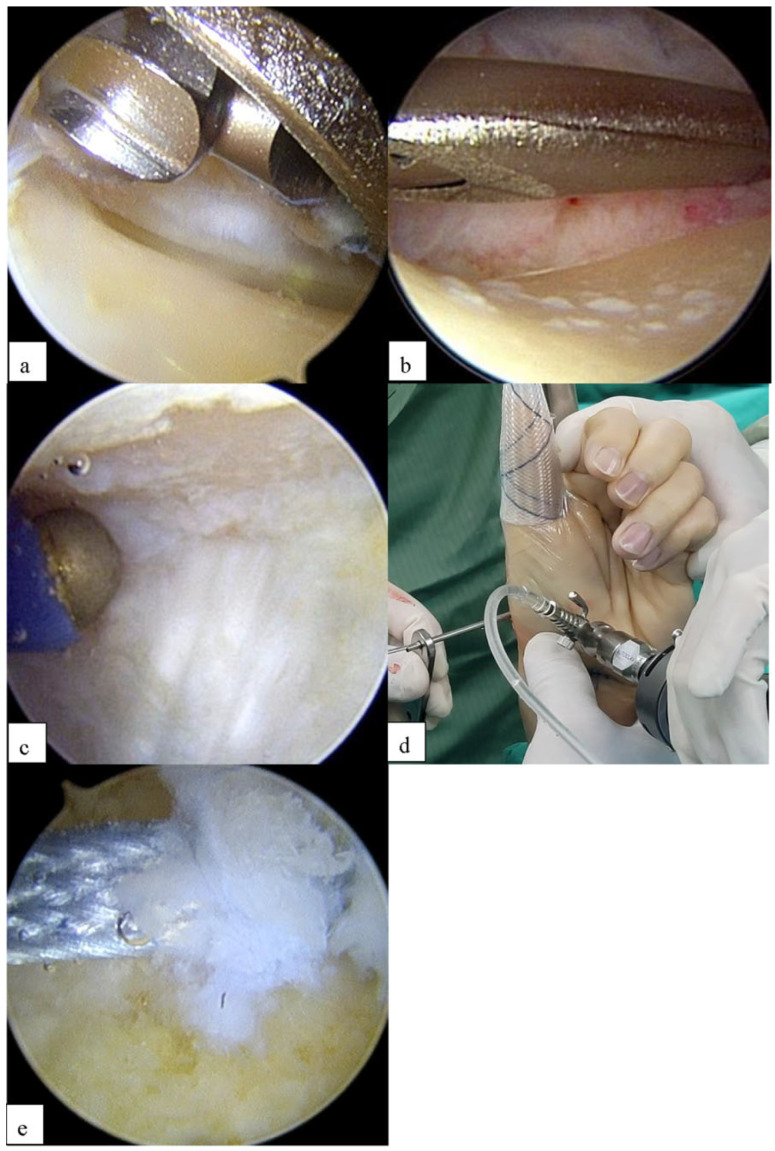
Arthroscopic evaluation and initial surgical steps for basal joint arthritis. (**a**) Arthroscopic view demonstrating stage III arthritis of basal joints. (**b**) Arthroscopic view showing one-third of the trapezium resected with power burr. (**c**) Thermal shrinkage of volar ligaments (beak) and capsule with RF probe. (**d**,**e**) Soft anchor (1.3 mm) applied to the base of the 2nd metacarpal region.

A full-radius mechanical shaver and a burr with suction were used in all patients for the initial debridement and partial trapeziectomy. All patients underwent arthroscopic synovectomy with a 2.0 mm shaver to remove any loose bodies or redundant capsules. The distal portion of the trapezium was excised using a 2.9 mm power burr, with approximately 3–5 mm of bone removed (Figure 3a,b). Ligamentous laxity and capsular attenuation were addressed using thermal capsulorrhaphy with an Arthrex CoolCut Ablator equipped with a small joint ball 45° probe (Ø 0.79 mm tip; Arthrex, Naples, FL, USA) [7]. This monopolar radiofrequency device is specifically designed for small-joint applications and provides controlled thermal energy delivery for precise tissue modification. The thermal shrinkage procedure was performed using a systematic approach to ensure uniform tissue contraction while avoiding thermal injury. The radiofrequency probe was gently applied to the volar beak ligament, joint capsule, and surrounding ligamentous structures using a controlled painting motion (Figure 3c). Constant probe movement was maintained to prevent excessive heat concentration in any single area, which could result in tissue necrosis or uncontrolled thermal damage. Radiofrequency energy was applied slowly and deliberately, allowing real-time visualization of tissue response including progressive tissue shortening and characteristic blanching. The thermal treatment continued until visible tissue contraction was achieved, indicating adequate collagen denaturation and subsequent tissue tightening. Throughout the procedure, meticulous care was taken to avoid direct contact with articular cartilage surfaces to prevent iatrogenic chondral damage. The ulnar portion of the articular surface articulating with the second metacarpal was also addressed during this thermal treatment phase. A 1.3 mm soft anchor (Conmed Y-knot Flex 1.3 mm W/one Ribbon tape, ConMed, Frenchs Forest, NSW, Australia) was arthroscopically applied to the base of the 2nd metacarpal under direct visualization (Figure 3d,e).

**Figure 4:**
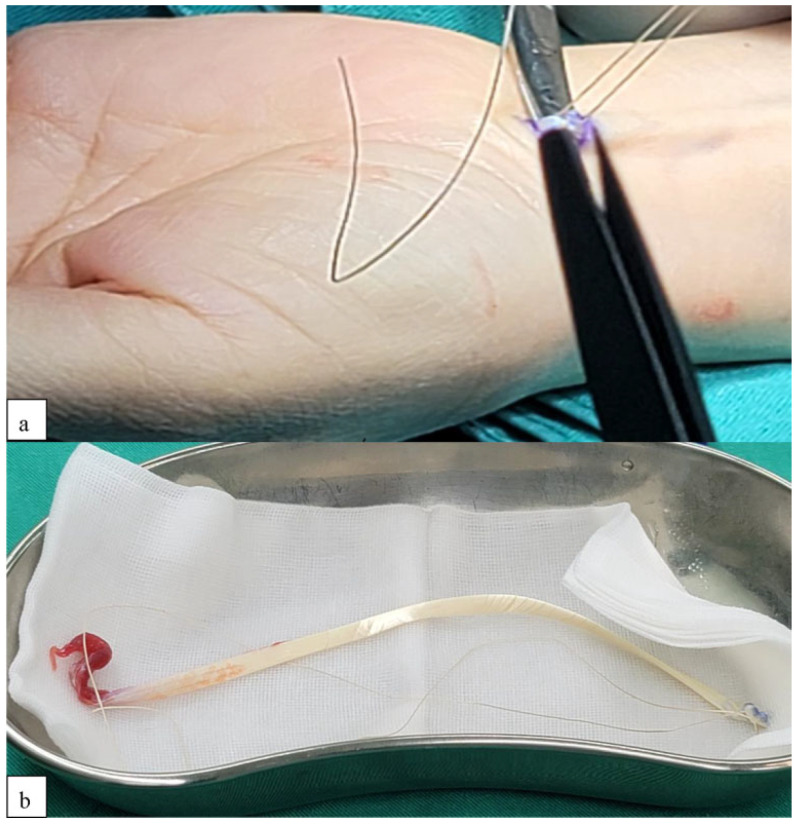
Tendon graft harvesting procedure. (**a**) PL tendon graft harvested from the volar forearm. (**b**) Free tendon graft taken from the same forearm site. (PL, palmaris longus.)

**Figure 5:**
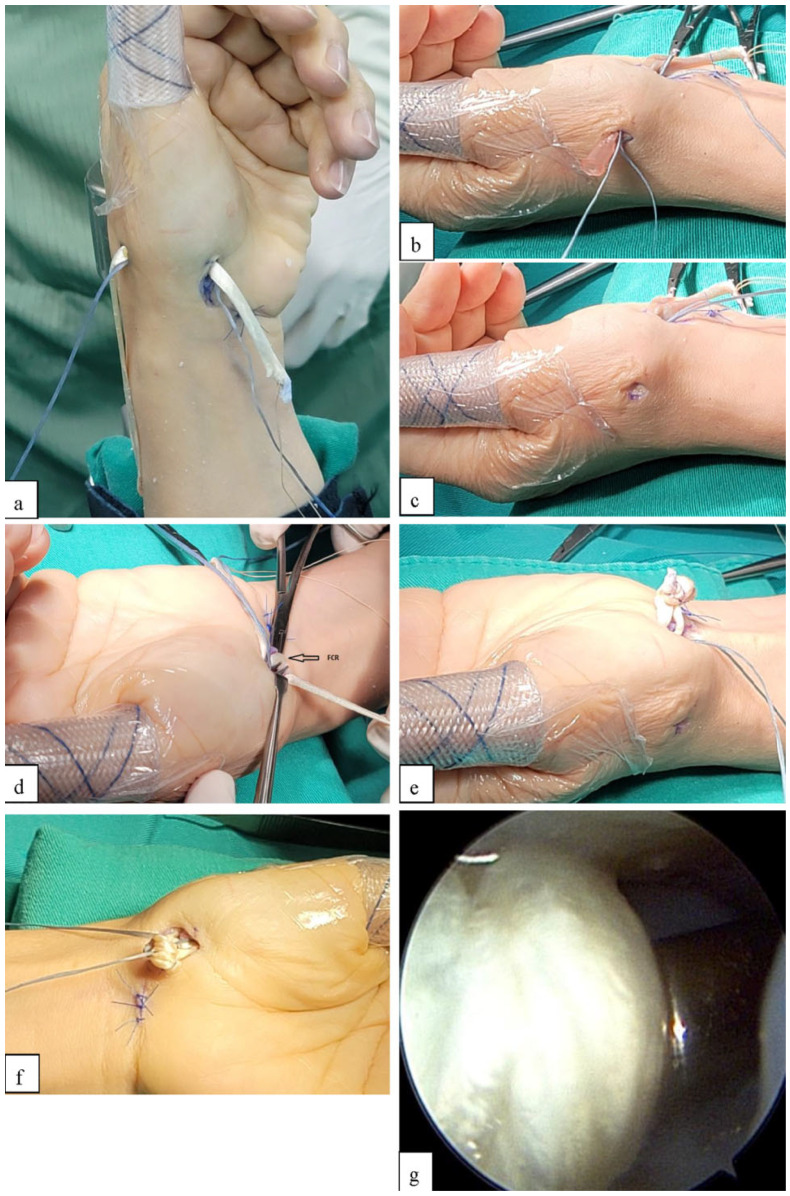
Tendon suspension and internal brace construction technique. (**a**) The PL tendon graft was passed across the joint from the thenar portal to the radial portal. One arm of the anchor was also pulled back to the radial portal. (**b**,**c**) The tendon graft was woven with the APL and passed back through the joint to the thenar portal. The anchor arm was also woven with the APL and returned to the thenar portal. (**d**) The PL tendon graft was woven with the FCR tendon and sutured in place. The soft anchor arm was also woven into the FCR tendon. (**e**) The residual tendon graft was fashioned into a tendon ball. (**f**) The remaining arm of the anchor was passed through the tendon ball, which was then pushed into the joint. (**g**) Arthroscopic view showing the tendon ball positioned in the basal joint and secured with the soft anchor. (PL, palmaris longus; APL, abductor pollicis longus; FCR, flexor carpi radialis).

The PL tendon was removed from the volar forearm using a tendon stripper (Figure 4). Under arthroscopy, the PL was passed through the basal joint from the thenar portal to the radial portal to increase the stability of the basal joint as part of the internal brace (Figure 5a). The PL tendon was woven with the APL tendon, and the joint was passed back to the thenar portal, while one arm of the 1.3 mm anchor was also woven with the APL and returned to the thenar portal (Figure 5b,c). The PL tendon graft was then woven with the FCR tendon, and the two were sutured together, while the arm of the soft anchor was woven with the FCR tendon (Figure 5d). The residual PL tendon graft was made into a tendon ball (Figure 5e); the other arm of the 1.3 mm anchor (Conmed Y-knot Flex 1.3 mm W/one Ribbon tape, ConMed, Frenchs Forest, NSW, Australia) was sutured through the tendon ball, and then the tendon ball was pushed into the joint (Figure 5f,g).

**Figure 6:**
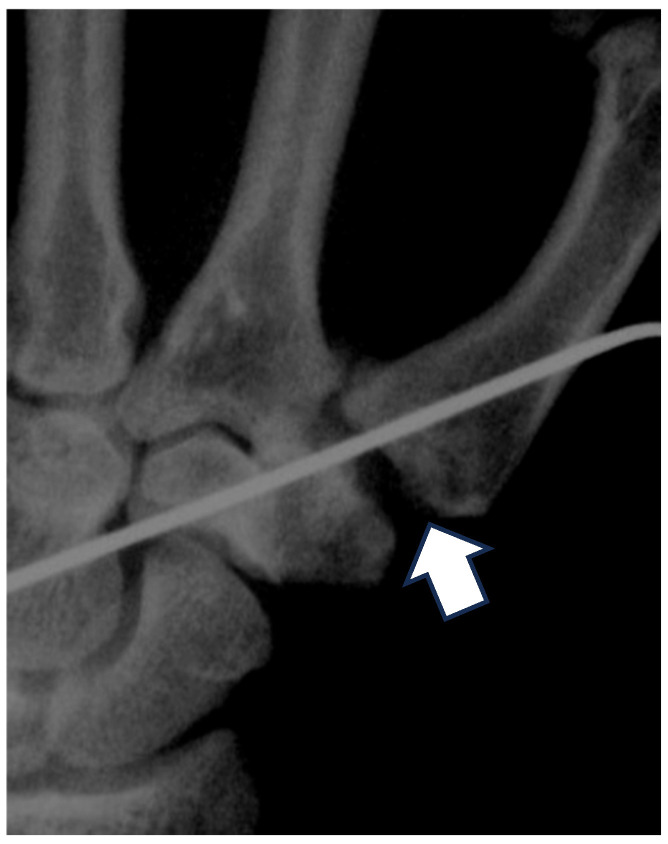
Basal joint fixed using a 1.2 mm K-wire for 4 weeks. Postoperative radiograph at 24-month follow-up showing maintenance of the arthroplasty space. The white arrow indicates the well-reserved space between the base of the first metacarpal and the remaining trapezium.

A 1.2 mm K-wire was passed percutaneously and longitudinally through the base of the metacarpal to the trapezial void to hold the base of the thumb metacarpal at the level of the trapezoid–index CMC joint (Figure 6). The wounds were closed with simple sutures, and a short-arm thumb pica splint was applied. Postoperatively, the sutures were removed at approximately 1 week, and a short-arm cast was applied for 4 weeks, at which point the percutaneous pin was removed. Formal occupational therapy was initiated, and a removable brace was worn for an additional 4 weeks.

**Figure 7:**
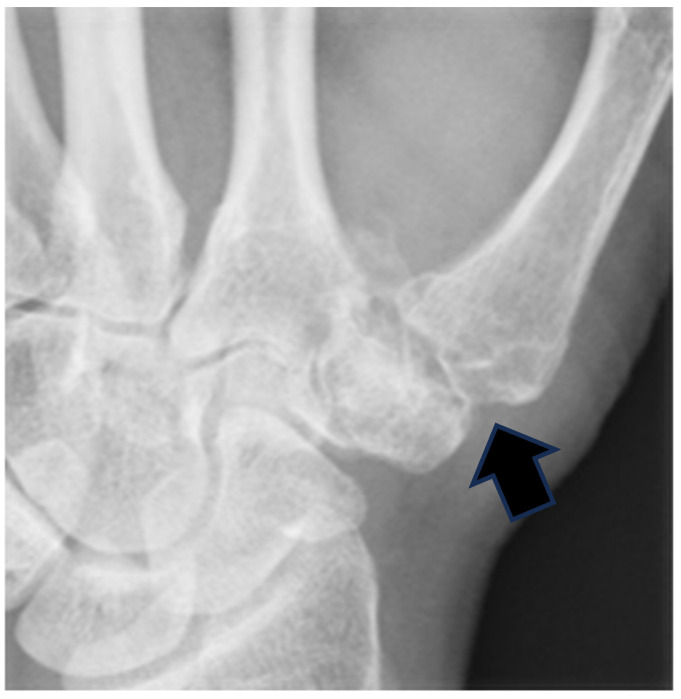
Postoperative radiograph at 2-year follow-up showing minimal proximal migration (1.2 mm) of the first metacarpal base. The black arrow indicates the preserved arthroplasty space between the base of the first metacarpal and the remaining trapezium.

Outcome assessments were performed using standardized protocols. Each patient underwent a comprehensive clinical evaluation preoperatively and at 24 months postoperatively. Pain was evaluated using a 10-point Visual Analog Scale (VAS), a validated tool for quantifying the subjective experience of pain [8,9]. The thumb’s range of motion (ROM) was measured using a standard goniometer, following American Society of Hand Therapists guidelines, which provides a reliable assessment essential for tracking functional changes [10]. Key pinch strength was assessed using a Jamar dynamometer (Therapeutic Equipment, Clifton, NJ, USA) according to the standardized protocols, with measurements recorded as a percentage of the contralateral side. This metric is particularly relevant, as a decreased key pinch strength correlates strongly with the severity of carpometacarpal osteoarthritis and overall hand functionality [11,12]. Standard anteroposterior and lateral radiographs were obtained at each follow-up to evaluate joint stability and potential subluxation or collapse (Figure 7). Basal joint space width was measured on radiographs immediately postoperatively and at 24 months to quantify joint collapse. All complications were systematically documented throughout the follow-up period. These assessment methods ensured consistent and reliable outcome measurements across all the study participants, aligning with established guidelines for evaluating hand surgery outcomes [13].

### 2.2. Statistical Analysis

Statistical analyses were performed using SPSS (version 22.0; IBM Corp., Armonk, NY, USA). Continuous variables were presented as mean ± standard deviation, while categorical variables were expressed as frequencies and percentages. The Shapiro–Wilk test was used to assess the normality of the data distribution. Demographic characteristics and baseline measurements between the Eaton stage II and III groups were compared using independent *t*-tests for continuous variables (age, VAS pain scores, thumb ROM, and pinch strength) and chi-square or Fisher’s exact tests for categorical variables (sex distribution and poor outcome rates). To evaluate the treatment’s effectiveness, paired *t*-tests were conducted to compare preoperative and postoperative measurements within each group, including VAS pain scores (at rest and during activity), thumb ROM, and pinch strength. Between-group differences in improvement (pre–post differences) were analyzed using independent *t*-tests. A poor functional outcome was defined as an improvement in pinch strength of less than 40% compared to the contralateral hand. Variables for the multivariate logistic regression analysis were selected based on clinical relevance and statistical significance in univariate analysis (*p* < 0.05). Before their inclusion in the multivariate model, a variance inflation factor (VIF) analysis was performed to check for multicollinearity among the variables, with VIF values > 5 considered to indicate significant multicollinearity. The final model included age, Eaton stage, preoperative VAS for thumb pain during activity, preoperative thumb ROM, and preoperative pinch power. A *p*-value < 0.05 was considered statistically significant for all analyses. No adjustments were made for multiple comparisons, as the analyses were exploratory in nature.

## 3. Results

This study included 60 patients with basal joint osteoarthritis, including 37 (61.7%) with Eaton stage II and 23 (38.3%) with Eaton stage III (Table 1). The overall mean age was 62.57 ± 4.31 years, with stage III patients (60.17 ± 3.31 years) being significantly younger than stage II patients (64.05 ± 4.22 years) (*p* < 0.001). Female patients predominated in both groups, accounting for 86.7% (52/60) of the total sample, with no significant difference in sex distribution between the groups (*p* = 1.000). In the preoperative assessment, stage III patients reported significantly higher VAS scores for thumb pain during activity (7.39 ± 0.50) than stage II patients (6.84 ± 0.50) (*p* < 0.001), while pain scores at rest showed no significant difference (*p* = 0.472). Preoperative thumb ROM and pinch strength (expressed as a percentage of the normal hand) did not differ significantly between groups, with overall means of 43.25 ± 11.31 degrees and 47.33 ± 9.50%, respectively. Notably, stage III patients demonstrated significantly greater collapse of the basal joint (2.35 ± 0.88 mm) than stage II patients (1.70 ± 0.57 mm) (*p* = 0.001). The mean follow-up time was 28.70 ± 2.95 months, with no significant difference between groups (*p* = 0.07). The proportion of patients with poor functional outcomes (pinch strength improvement < 40%) was significantly higher in stage III patients (39.1%) than in stage II patients (8.1%) (*p* = 0.006) (Table 1).

All patients showed significant functional improvement following surgery. VAS scores for thumb pain at rest decreased from 5.68 ± 0.47 preoperatively to 0.98 ± 0.70 postoperatively (*p* < 0.001), with a significant difference in improvement between groups (*p* = 0.008), where stage II patients showed greater improvement than stage III patients (−4.89 ± 0.77 vs. −4.39 ± 0.50) (Table 2). VAS scores for thumb pain during activity decreased from 7.05 ± 0.57 preoperatively to 1.35 ± 0.92 postoperatively (*p* < 0.001), with no significant difference in improvement between groups (*p* = 0.786). Thumb ROM increased from 43.25 ± 11.31 degrees preoperatively to 54.17 ± 9.75 degrees postoperatively (*p* < 0.001), with stage II patients showing significantly greater improvement (12.84 ± 9.54 degrees) than stage III patients (7.83 ± 6.18 degrees) (*p* = 0.029). Pinch strength (as a percentage of the normal hand) improved from 47.33 ± 9.50% preoperatively to 88.83 ± 17.33% postoperatively (*p* < 0.001). Although stage II patients showed a numerically greater improvement (43.65 ± 7.61%) than stage III patients (38.04 ± 19.17%), the difference did not reach statistical significance (*p* = 0.116) (Table 2).

The logistic regression analysis identified the factors associated with a poor postoperative functional recovery (pinch strength improvement < 40%). In the univariate analysis, Eaton stage III (OR = 7.29, 95% CI: 1.67–28.82, *p* = 0.008), higher preoperative VAS scores for thumb pain during activity (OR = 7.86, 95% CI: 2.20–30.12, *p* = 0.004), and lower preoperative thumb ROM (OR = 0.92, 95% CI: 0.86–0.99, *p* = 0.023) were significantly associated with poor functional recovery (Table 3). In the multivariate analysis, after adjusting for potential confounding factors, Eaton stage III (OR = 10.28, 95% CI: 2.47–26.6, *p* = 0.012), higher preoperative VAS scores for thumb pain during activity (OR = 11.48, 95% CI: 1.38–40.23, *p* = 0.038), and lower preoperative thumb ROM (OR = 0.69, 95% CI: 0.52–0.93, *p* = 0.015) remained independent risk factors for poor functional recovery. Age and pre-operative pinch strength were not significantly associated with poor functional recovery (*p* > 0.05). Due to the small number of male patients, sex could not yield a valid OR estimate in the univariate analysis (Table 3).

No infections or immediate complications were recorded in any patient who underwent the procedure. All patients returned to work or resumed daily activities within 3 months. By radiographic follow-up, all patients had approximately one-half to one-third of the distal trapezium resected, and this resected space decreased by only 1.1 mm (range: 0–4 mm) over 3 months. None of the patients had basal joint subluxation or STT joint arthritis.

## 4. Discussion

Arthroscopy is a reliable method for evaluating and treating thumb CMC joint arthritis [5,14,15,16]. Culp and Rekant were the first clinicians to suggest evaluation, debridement, and synovectomy. Some studies also demonstrated good results when the instability of the thumb CMC joint is treated with thermal capsulorrhaphy and volar ligamentous shrinkage using a radiofrequency shrinkage probe [15]. In our procedure for the arthroscopic treatment of basal joint arthritis, we performed a thermal ligamentous and capsular shrinkage of the superficial and deep AOL ligament, ulnar collateral ligament, posterior oblique ligament, and capsule after partial trapeziectomy [7]. The integrity of the AOL has been emphasized in many studies as being important for maintaining the stability of the CMC joint. The capsular thermal procedure also involves a partial neurectomy, because it destroys afferent sensory receptors [16]. To recreate functional volar ligaments, researchers have used several methods of ligament repair or reconstruction. These are technically challenging procedures with various complications. Therefore, the use of electrothermal shrinkage techniques to treat volar ligament laxity is appealing. Electrothermal methods enable the treatment of patients with volar ligament instability with minimal complications. We used a 1.9 mm arthroscopy to perform this procedure in patients of East Asian ethnicity. These patients prefer a procedure with a quick recovery period that is less invasive and painful than traditional methods. The use of small-joint arthroscopy in the smaller joints of the hand has been discussed and refined over the years by other authors [17,18,19,20].

An arthroscopic-free PL tendon suspension and interposition arthroplasty are per-formed to effectively debride or replace irregular articular surfaces. In our procedure, the PL tendon was taken, passed through the basal joint, and tightly weaved with the FCR and APL tendons, which should enhance the stability of the first metacarpal base and prevent subsiding or radial subluxation [21,22]. It is important to pack the joint space as much as possible to keep the first metacarpal and residual trapezium apart. The interpositions also maintain the stability of the basal joints to preserve their space. In our study, most patients had a good stability and preserved space in the basal joint on subsequent radiographs. The amount of additional height loss that occurs under dynamic loading remains unknown. In our study, a loss of height greater than 20% was only noted in 3 of 23 (13.0%) patients. Gortex was used as an interpositional material, because it is readily available, saves time, and avoids the need for another incision to harvest the tendon [23]. Recently, many studies have shown that Gortex can cause particulate diseases and has, therefore, not been recommended as an interpositional material [24]. The best material for this procedure is an autogenous tendon graft. Bleeding from the subchondral bone fills the interspaces of the coiled tendon grafts. The clot organizes and is eventually converted into fibrous tissue, which acts as a spacer, keeping the opposing bones apart.

During the arthroscopic partial trapezial resection, we could locate the second metacarpal base from the basal joint. A 1.3 mm ribbon tape soft anchor was applied at the base of the second metacarpal. One limb of the anchor was sutured through the insertion of the APL tendon and then back to the thenar portal, followed by tightening with another limb to further increase the stability of the thumb through the internal brace. We believe that the accurate and reproducible measurement of metacarpal migration is the ultimate proof of the stability of a basal joint arthroplasty construct. The radiographic technique presented here appears to provide a precise means of quantifying proximal metacarpal migration by comparing the preoperative height ratios obtained at the latest postoperative follow-up visit. Dorsal migration of the metacarpal base is minimal because of the anatomic restoration of volar stabilization using thermal shrinkage of the volar ligaments, tendon suspension, and an internal brace. Total excision of the trapezium is recommended, because the peritrapezial joints are almost always involved when the basal joint is affected, even during the early stages. This point was not substantiated by cadaveric studies conducted by Eaton [25]. They found that the STT joint was involved in approximately 47% of cases, along with the trapeziometacarpal joint, and routine trapeziectomy was unnecessary. Total excision of the trapezium resulted in the proximal migration of the first metacarpal and a weak pinch [26]. It also destroyed the STT joint and caused instability of the scapholunate joint. To avoid this, many surgeons have elected to remove just the articular surface of the trapezium [27,28,29]. In our study, no patient developed postoperative arthritis of the scaphotrapezial joint. Only an average of a 1.2 mm (range 0.8–2.6) migration of the first metacarpal bone was found in the 2-year follow-up.

Our findings align with the broader surgical literature on basal joint osteoarthritis. Mathoulin et al. reported that ligamentoplasty combined with trapeziectomy yielded better outcomes in earlier disease stages [30]. Similarly, Gibbons et al. and Raven et al. demonstrated higher satisfaction rates and fewer complications following trapeziectomy in stage II patients compared to advanced cases [31,32]. Kemper et al. found that arthroscopic techniques produced significant functional improvements that diminished in advanced stages, consistent with our observation that 39.1% of stage III patients experienced a poor functional recovery (pinch strength improvement < 40%) compared to just 8.1% in stage II [33]. As osteoarthritis progresses, anatomical changes, including joint collapse and ligament degradation, complicate surgical interventions and reduce the recovery potential [34]. Our study reinforces the importance of addressing basal joint arthritis in earlier stages to optimize clinical outcomes.

Our findings show that Eaton stage II patients demonstrated better functional outcomes than stage III patients, aligned with the natural progression of basal joint arthritis. In stage II disease, the articular cartilage damage is less extensive, and there is minimal joint space narrowing compared to stage III, where significant cartilage loss, subchondral sclerosis, and osteophyte formation are present. The more advanced degenerative changes in stage III likely result in a greater disruption of normal joint biomechanics and more extensive soft tissue contractures. Our arthroscopic technique, while effective for both stages, may be particularly beneficial for stage II patients, where preservation of the remaining healthy joint structures and addressing ligamentous laxity can more effectively restore normal joint function. This finding suggests that earlier surgical intervention, before progression to more advanced disease, may be advantageous for optimizing outcomes. Recent literature supports this observation. Carità et al. and Edwards & Ramsey have documented that stage III involves significant joint destruction and subluxation, leading to an irreversible loss of anatomical integrity, whereas stage II presents with less severe degenerative changes and better functional preservation [35,36]. Wilkens et al. demonstrated that arthroscopic techniques yield more consistent outcomes in patients with early-stage arthritis (stages I and II) compared to advanced stages [37]. Furthermore, management strategies for stage III often require more invasive surgical options, such as arthrodesis or total joint arthroplasties, rather than the joint-preserving techniques suitable for stage II patients [38,39]. These more invasive procedures are associated with higher risks of complications that can adversely affect recovery, including stiffness and a reduced range of motion [40]. The structural deterioration in stage III can lead to diminished muscle function and strength, as reflected in a decreased grip strength and functional capacity compared to stage II counterparts [41,42]. Our results confirm these observations from the literature, highlighting the importance of disease staging in predicting functional outcomes after surgical intervention.

Alternative emerging treatments for basal joint arthritis include suspensionplasty using suture-button devices, trapeziometacarpal joint arthrodesis, and various implant arthroplasties. Suture-button suspensionplasty, as described by Yao et al., involves complete trapeziectomy followed by suspension of the first metacarpal using a suture-button construct between the first and second metacarpals [43]. Their study of 21 patients reported a mean DASH score improvement from 52 to 15 at 24 months, with first metacarpal subsidence averaging 2.1 mm. Compared to our technique, suture-button suspensionplasty still requires complete trapezium removal and does not address ligamentous laxity through thermal shrinkage. Our approach demonstrated less proximal migration and preserved the STT joint, potentially offering better long-term stability while achieving comparable pain relief and functional improvement. Hemiresection interposition arthroplasty (HIA) has also gained attention as an alternative approach. Spiteri and Giele conducted a systematic review comparing various surgical techniques for CMC joint osteoarthritis and found that hemiresection with interposition materials provided good pain relief and functional improvement while requiring less soft tissue dissection [44]. This contributes to preserving joint stability and maintaining the thumb’s length and strength, particularly in patients with Eaton stage II and early III osteoarthritis. Maio et al. noted that while traditional trapeziectomy with ligament reconstruction showed long-term benefits, it could leave patients with residual instability—a complication that HIA aims to mitigate through interposition grafts [45]. Our arthroscopic technique shares some principles with HIA, particularly the preservation of part of the trapezium, but it adds the benefits of minimal invasiveness, thermal shrinkage for ligamentous stabilization, and internal bracing for additional support. While complete trapeziectomy with LRTI remains the standard for advanced basal joint arthritis, our technique and other partial preservation approaches may offer advantages for earlier disease stages with potentially fewer complications.

The present study has several limitations. First, its retrospective design, without a control group, limits our ability to directly compare our arthroscopic technique with traditional open procedures. A prospective randomized controlled trial comparing arthroscopic partial trapeziectomy with an internal brace to conventional LRTI would provide stronger evidence of its comparative efficacy. Second, our follow-up period of approximately 28 months, which is sufficient to demonstrate short-to-mid-term outcomes, may not have captured potential long-term complications or deterioration. Future studies with a 5–10-year follow-up period would be valuable to assess the durability of the procedure and monitor for late complications, such as progressive metacarpal subsidence or the development of STT arthritis. Third, the assessment of functional outcomes primarily focused on pain and strength measurements. A more comprehensive evaluation using validated hand function questionnaires, such as the Disabilities of the Arm, Shoulder, and Hand or the Michigan Hand Outcomes Questionnaire, would provide more robust patient-reported outcome data. Fourth, our logistic regression analysis identified three independent risk factors for poor functional recovery: Eaton stage III disease, higher preoperative pain scores during activity, and a lower preoperative thumb ROM. While these findings aligned with previous studies suggesting that more advanced disease and greater preoperative impairment are associated with poorer outcomes, the confidence intervals for some odds ratios are relatively wide. This statistical limitation reflects our modest sample size of 60 patients, which may affect the precision of our estimates. Nevertheless, the consistency of these risk factors across both univariate and multivariate analyses provides some confidence in the reliability of our findings. Future studies with larger cohorts would be valuable to confirm these associations with greater statistical power and narrower confidence intervals.

Future research directions should include biomechanical studies to quantify the stabilizing effect of the internal brace construct compared to traditional suspension techniques; comparative imaging studies using advanced modalities such as MRI or CT to better characterize the maintenance of the arthroplasty space over time; an investigation of potential modifications to the technique for patients with advanced disease (Eaton stage III), who demonstrated less favorable outcomes in our study; and an exploration of adjunctive biological treatments such as platelet-rich plasma or stem cell applications to potentially enhance tissue healing and functional recovery.

Despite these limitations, the proposed technique has several advantages. This method is less invasive than conventional open surgery. Arthroscopic surgery of the basal joint can detect articular cartilage damage long before radiological changes become evident. The capsule is not destroyed, thus maintaining a closed space for a true soft-tissue suspension and interpositional arthroplasty. Visualization through this surgical technique is excellent, including the medial site of the basal joint, and spurs can also be removed with a power burr. Thus, there is a lower chance of injury to the radial nerves. It is an effective procedure for treating stage II–III basal joint arthritis.

## 5. Conclusions

Arthroscopic partial trapeziectomy with tendon suspension/interposition and internal brace augmentation is an effective treatment for Eaton stage II–III basal joint arthritis. This minimally invasive technique provides significant pain relief and functional improvement, preserves the STT joint, minimizes metacarpal migration, and offers enhanced stability with fewer complications than traditional methods.

## Figures and Tables

**Table 1:** Demographic and clinical characteristics of patients undergoing arthroscopic partial trapeziectomy with tendon suspension/interposition and internal brace (*n* = 60).

	Eaton Stage		Total	*p*-Value
	3	2		
*n*	23	37	60	
Age	60.17 ± 3.31	64.05 ± 4.22	62.57 ± 4.31	<0.001 *
Sex (%)				1.000
Male	3 (13.0%)	5 (13.5%)	8 (13.3%)	
Female	20 (87.0%)	32 (86.5%)	52 (86.7%)	
PreOP VAS for thumb pain (rest)	5.74 ± 0.45	5.65 ± 0.48	5.68 ± 0.47	0.472
PreOP VAS for thumb pain (activity)	7.39 ± 0.50	6.84 ± 0.50	7.05 ± 0.57	<0.001 *
PreOP thumb ROM (°)	45.43 ± 10.54	41.89 ± 11.69	43.25 ± 11.31	0.241
PreOP pinch power (% of normal hand)	44.78 ± 11.63	48.92 ± 7.65	47.33 ± 9.50	0.101
Progress of pinch power (% of normal hand)	38.04 ± 19.17	43.65 ± 7.61	41.50 ± 13.41	0.116
Poor outcome (progress < 40%) (%)	9 (39.1%)	3 (8.1%)	12 (20.0%)	0.006 *
Collapse of basal joint (mm)	2.35 ± 0.88	1.70 ± 0.57	1.95 ± 0.77	0.001 *
Follow-up time (M)	27.83 ± 1.83	29.24 ± 3.38	28.70 ± 2.95	0.070

Data are presented as *n* (%) or mean ± standard deviation. * *p*-value < 0.05 was considered statistically significant after test.

**Table 2:** Postoperative recovery of functional outcome (*n* = 60).

Item	Eaton Stage	*n*	Pre-OP	Post-OP	Difference	Within-Group *p*-Value	Between-Group *p*-Value
VAS for thumb pain (rest)	3	23	5.74 ± 0.45	1.35 ± 0.49	−4.39 ± 0.50	<0.001 *	0.008 *
	2	37	5.65 ± 0.48	0.76 ± 0.72	−4.89 ± 0.77	<0.001 *	
	Total	60	5.68 ± 0.47	0.98 ± 0.70	−4.70 ± 0.72	<0.001 *	
VAS for thumb pain (activity)	3	23	7.39 ± 0.50	1.74 ± 0.86	−5.65 ± 1.03	<0.001 *	0.786
	2	37	6.84 ± 0.50	1.11 ± 0.88	−5.73 ± 1.10	<0.001 *	
	Total	60	7.05 ± 0.57	1.35 ± 0.92	−5.70 ± 1.06	<0.001 *	
Thumb ROM (°)	3	23	45.43 ± 10.54	53.26 ± 12.21	7.83 ± 6.18	<0.001 *	0.029 *
	2	37	41.89 ± 11.69	54.73 ± 7.99	12.84 ± 9.54	<0.001 *	
	Total	60	43.25 ± 11.31	54.17 ± 9.75	10.92 ± 8.71	<0.001 *	
Pinch power (% of normal hand)	3	23	44.78 ± 11.63	82.83 ± 21.63	38.04 ± 19.17	<0.001 *	0.116
	2	37	48.92 ± 7.65	92.57 ± 13.00	43.65 ± 7.61	<0.001 *	
	Total	60	47.33 ± 9.50	88.83 ± 17.33	41.50 ± 13.41	<0.001 *	

Data are presented as mean ± standard deviation. * *p*-value < 0.05 was considered statistically significant after test.

**Table 3:** Factors associated with poor postoperative functional recovery (<40%) (*n* = 60).

	Crude		Adjusted	
	OR (95% CI)	*p*-Value	OR (95% CI)	*p*-Value
Age	0.99 (0.86, 1.15)	0.952	1.31 (0.87, 1.99)	0.200
Eaton stage (3 vs. 2)	7.29 (1.67, 28.82)	0.008 *	10.28 (2.47, 26.6)	0.012 *
PreOP VAS for thumb pain (activity)	7.86 (2.20, 30.12)	0.004 *	11.48 (1.38, 40.23)	0.038 *
PreOP thumb ROM (°)	0.92 (0.86, 0.99)	0.023 *	0.69 (0.52, 0.93)	0.015 *
PreOP pinch power (% of normal hand)	0.97 (0.90, 1.04)	0.341	1.10 (0.92, 1.33)	0.303

Data are presented as odds ratio (95% CI). * *p*-value < 0.05 was considered statistically significant after test.

## Data Availability

All the data generated are within this article.

## Abbreviations

The following abbreviations are used in this manuscript:

APL	Abductor pollicis longus
CMC	Carpometacarpal
DASH	Disabilities of the Arm, Shoulder, and Hand
EPB	Extensor pollicis brevis
FCR	Flexor carpi radialis
LRTI	Ligament reconstruction and tendon interposition
PL	Palmar longus
ROM	Range of motion
RP	Radial portal
STT	Scaphotrapezial
TLA	Three-letter acronym
TP	Thenar portal
